# Creation of a Virtual Reality Telesimulation Program in Response to Mandatory COVID-19 Social Distancing During the Pandemic: A Primer for Those considering VR Simulation and Application to a Group of Physicians Naive to Virtual Reality

**DOI:** 10.1089/jmxr.2024.0027

**Published:** 2024-07-30

**Authors:** Nicholas Slamon, Odiraa Nwankwor, Kimberly Canter, Amanda Lewis, Anuradha Setlur, Jennifer Lutz

**Affiliations:** 1 Department of Pediatric Critical Care Medicine, Nemours Children’s Hospital Delaware, Wilmington, Delaware, USA.; 2 Department of Pediatric Psychology, Nemours Children’s Hospital Delaware, Wilmington, Delaware, USA.; 3 Department of Biostatistics and Research, Nemours Children’s Health, Wilmington, Delaware, USA.; 4 Department of Pediatric Critical Care Medicine, Miller Children’s Hospital Long Beach, Long Beach, California, USA.; 5 Department of Pediatric Critical Care Medicine, Lehigh Valley Reilly Children’s Hospital, Allentown, Pennsylvania, USA.

**Keywords:** pediatrics, pediatric critical care, virtual reality simulation, telesimulation, COVID-19

## Abstract

The COVID-19 pandemic necessitated the closure of traditional simulation centers, prompting innovative solutions for medical education. Drawing from prior studies, which advocated for telesimulation and virtual reality (VR) as alternatives, this article explores the development and implementation of VR simulation in medical training. Leveraging the Acadicus^®^ VR platform, a VR simulation solution was created, enabling interactive scenarios simulating pediatric critical care situations. Thirty-one diverse scenarios were designed and executed over an 8-month period, involving pediatric and emergency medicine residents and fellows. The development process involved creating lifelike mannequins and dynamic cardiac waveforms, enhancing realism and spontaneity. Using VR headsets and streaming technology, participants engaged in immersive scenarios remotely. Performance evaluation used a modified version of the Tool for Resuscitation Assessment Using Computer Simulation, revealing comparable outcomes across different training levels and specialties. Participant feedback underscored the immersive nature of VR simulation, offering enhanced realism and in-depth debriefing opportunities compared with traditional mannequin-based simulation. However, limitations such as the lack of haptic feedback and the need for better integration with existing simulation center infrastructure were noted. Cost-effectiveness emerged as a significant advantage of VR simulation, with lower upfront costs compared with traditional simulation centers. VR simulation also demonstrated versatility in staging training across various hospital settings, offering a more comprehensive learning experience. Although acknowledging the need for further research to measure skill acquisition and retention, this study highlights the potential of VR simulation as an adjunctive modality in medical education.

## Introduction

The closure of simulation centers during the COVID-19 (novel coronavirus SARS-CoV-2) pandemic resulted in medical education innovations. The 2020 paper by Wagner and Gross et al. studied simulation during pandemics and other disasters. They concluded, “in the future, new technology such as tele simulation or virtual reality might offer a method to continue education and training while maintaining social distance.”^1^ Centers instituted “distance learning” by streaming mannequin simulation via platforms such as Zoom Video Communications Inc. (San Jose, California). A study entitled “The state of distance healthcare simulation during the COVID-19 pandemic: results of an international survey” evaluated responses from 32 countries with 618 respondents: 70% of respondents reported that their simulation center was conducting distance simulation and 82% indicated long-term plans for maintaining a hybrid format relative to going back to in-person simulation (11%, *p* < 0.001).^2^ Although this may not have been optimal for team training and certainly affected the ability of learners to practice hands-on tasks such as cardiopulmonary resuscitation (CPR), many felt that telesimulation fit under the category of the old English proverb “something is better than nothing.” Learners directed teams of masked simulation staff to execute the steps of the scenario remotely while assisted by others watching the same live stream.

Drawbacks included difficulties in closed-loop communication via video and the need for creative ways to debrief with those not onsite. Quality of a single camera view was another challenge. Staff had to prepare remote simulation adjuncts such as radiographs, laboratory results, and debriefing charts. Finally, telesimulation was limited by less than lifelike plastic mannequins and the inability to change the surroundings to simulate different hospital areas. A 2021 paper by Diaz and Walsh during the pandemic echoed these drawbacks. “Educators and facilitators must understand the nuances between tele simulation and traditional simulation-based education prior to delivering a tele simulation-based program. In addition, this modality requires practice. Educators need to be facile with and effectively troubleshoot technology so that the participants are able to focus on learning rather than technical mishaps.”^3^

Virtual reality (VR) technology in the healthcare setting is growing. Chang et al. described at least four current uses for VR. Two are patient facing and based in relaxation, therapeutic escape, and desensitization. They are used primarily in patients undergoing repetitive stressful interventions such as intravenous catheter access or chemotherapy/radiation. Provider facing VR examples include three-dimensional (3D) visualization for complex operative modeling and team training/simulation.^4^ More recently, VR expanded into including patient education for complex diseases like diabetes,^5^ as an adjunct to physical and occupational therapies via game play,^6^ and in remote telepsychiatry and counseling.^7^

By leveraging VR technology at our institution, we were able to build upon the idea of distance or “telesimulation” and augment the experience beyond traditional mannequin simulation. Moreover, the combination of VR simulation with live video distance simulation was novel.

## Methods

The aims of this project were two-fold and separated in time by 7 months. First, the group addressed the need for continued medical education during the COVID-19 shutdown of physical simulation centers through the creation of a VR simulation solution. The second aim was to then deploy the VR simulation to a wide group of physician learners in the pediatric intensive care unit (PICU) and report the findings associated with this new method of medical education.

### VR simulation development

**FIG. 1.**
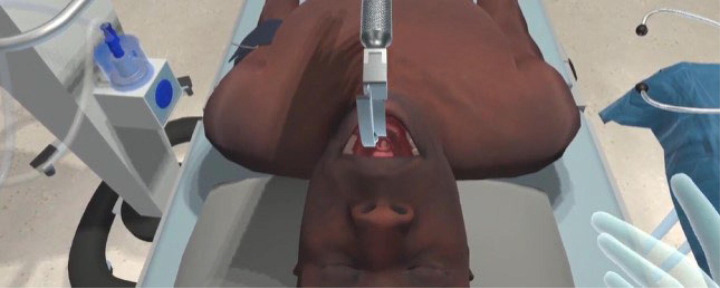
Teen mannequin intubation sequence.

Creation of the VR simulation solution began in November 2020, 6 months after the start of COVID-19 shutdowns in the United States. A member of the study group had previous experience with a VR platform, Acadicus^®^ (Madison, Wisconsin) in the design of an intubation education tool. The application is an expansive “digital sandbox” of virtual content for creating multiplayer simulations and 3D-recorded education. The shared creation model of the platform allowed access to and editing of previous content to design novel simulation scenarios. Environments such as a digital twin of an eight-bay intensive care unit and working models of key equipment such as laryngoscopes, catheters, and ventilators, allowed dedication of the entire budget ($25,000, Nemours Foundation Grant) to the purchase of two laptops and headsets, the development of three interactive mannequins and common cardiac waveforms. An infant, a school-age child, and a teenager covered a broad range of pediatric disease processes. Each mannequin possessed the ability to breathe, seize, and had intravenous catheter insertion sites located in the arm, hand, or foot. In addition, bag-mask ventilation, intubation (as shown in Fig. 1), and pleural catheter insertion, could be demonstrated through a sequence of animated steps.

**FIG. 2.**
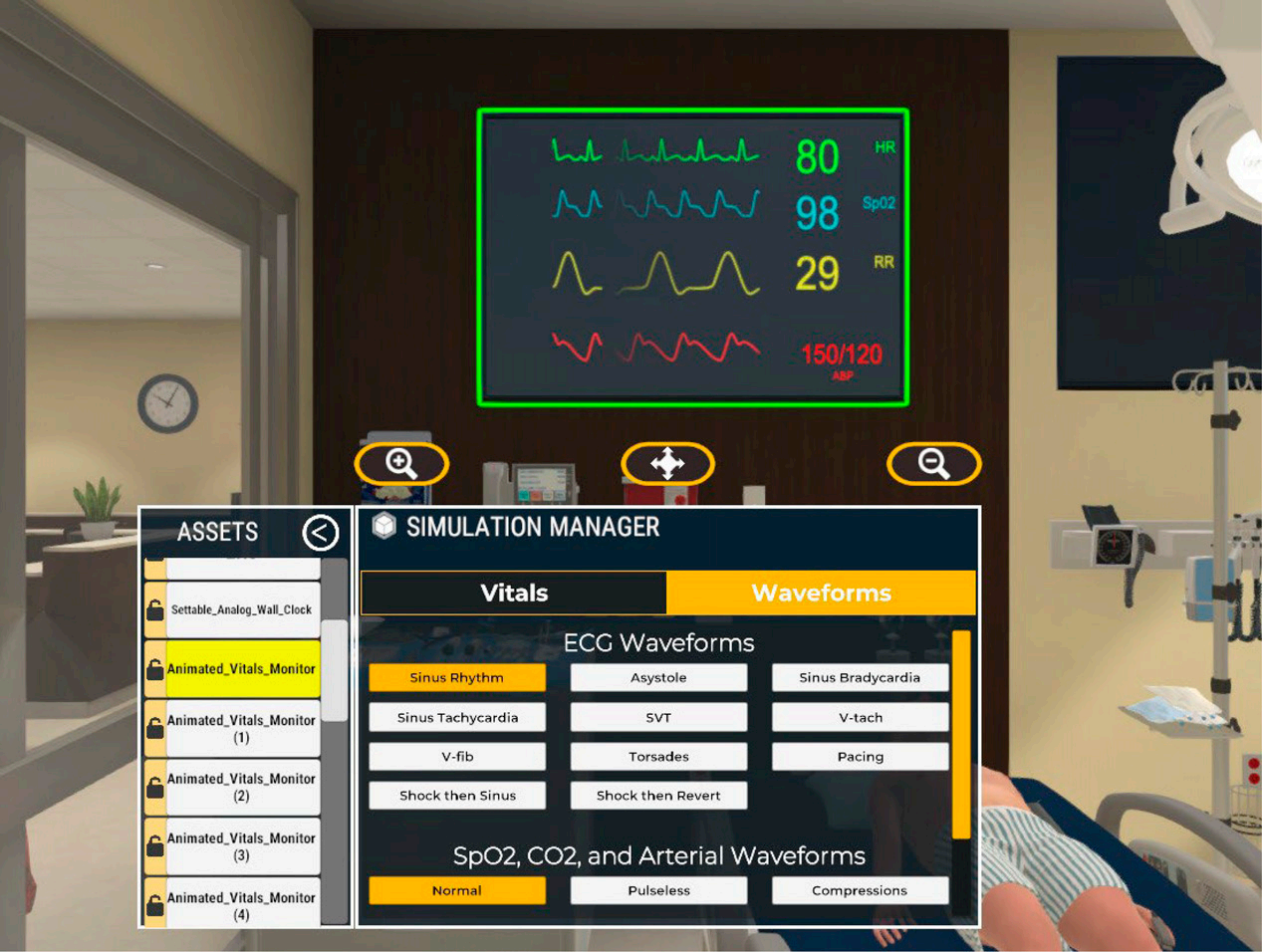
Simulation manager waveform options.

A series of realistic continuous cardiac waveforms to represent the common arrhythmias encountered in pediatric advanced life support (PALS) or advanced cardiac life support (ACLS) training were created. A central control panel or “simulation manager” allowed rhythm and vital sign change in real time with a simple button push by study members to maximize the realism and spontaneity. Additional rhythm choices included sinus tachycardia, supraventricular tachycardia, ventricular fibrillation and tachycardia, sinus bradycardia, asystole, Torsade’s de pointes as well as pacing, defibrillation, pulselessness, and compressions (Fig. 2).

**FIG. 3.**
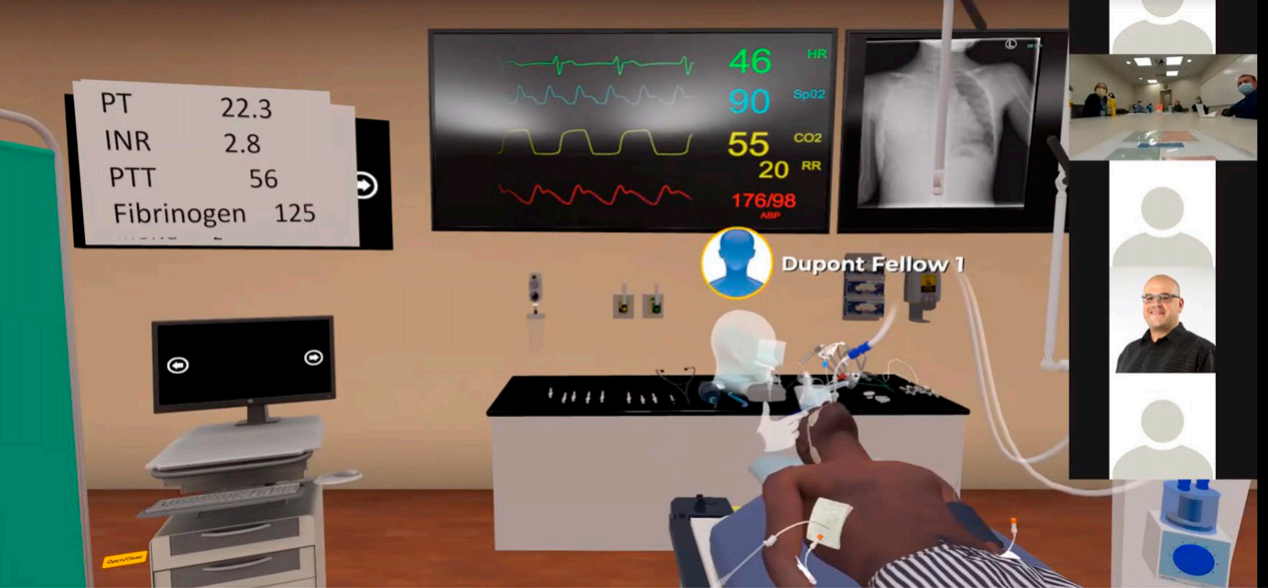
Multitrauma victim scenario.

A portion of the budget was dedicated to the purchase of two gaming laptops (Alienware^®^, Dell Inc., Miami, Florida) and two Oculus Rift S^®^ headsets (Lenovo Inc., Morrisville, North Carolina). Two laptops allowed for two members of the study group to act from within the VR scenario as avatars who could execute the instructions of the code leader who was viewing the scenario live and remotely via Zoom^®^. A third laptop served as the streaming device in a fixed view from the traditional code leader position in the virtual patient room (Fig. 3).

### Scenario design

A series of scenarios that would be challenging to emergency medicine, pediatric residents, and pediatric critical care fellows were created. Thirty-one different cases were simulated over an eight-month period from June 2021 through January 2022 as shown in Table 1. A series of adjunctive learning tools for each scenario. These included static deidentified images such as chest radiographs as well as dynamic studies like echocardiograms and ultrasounds. Flow charts, graphics, and additional key assets to enhance realism were also added. Examples included modification of existing Acadicus assets such as noninvasive mechanical ventilation equipment like bilevel positive airway pressure (BiPAP) masks that were resized to fit pediatric sized virtual patients and the novel creation of all the supplies needed to insert a thoracostomy (chest) tube in the newly created virtual mannequins (Table 2).

**Table 1.** VR Simulation Scenarios

Simulation scenario	Case description
1	7-year-old male ex-25-week preterm infant, asthma, supraventricular tachycardia while on continuous albuterol
2	9-day-old female with congenital adrenal hyperplasia, hyperkalemia, and ventricular tachycardia arrest
3	2-week-old male with upper respiratory infection, right mainstem intubation, and L-sided collapse with hypoxia
4	15-year-old male with bacterial pneumonia, large R pleural effusion for chest tube placement secondary to desaturation
5	6-year-old with B cell acute lymphocytic leukemia, fever, sepsis, and seizure due to meningitis
6	14-year-old male with subdural hematoma and altered mental status after all terrain vehicle (ATV) rollover, elevated intracranial pressure
7	6-year-old with supraventricular tachycardia that degenerates into ventricular arrhythmia
8	16-year-old with R-sided hemothorax following motor vehicle accident requiring chest tube and resuscitation
9	2-month-old infant with neonatal seizures
10	8-year-old male with status asthmaticus
11	9-year-old medically complex child with tracheostomy and tracheal bleeding
12	8-year-old with rhinoviral infection, wheezing, and supraventricular tachycardia after initiation of albuterol
13	14-year-old with tension pneumothorax, Displacement, Obstruction, Pneumothorax or Equipment failure mnemonic scenario in an intubated patient
14	6-month-old with Respirator syncytial virus (RSV) bronchiolitis and impending respiratory failure
15	8-year-old female with cardiac arrest secondary to myocarditis
16	7-year-old medically complex male with bradycardia following failed endoscopic third ventriculostomy for hydrocephalus
17	13-year-old with multiple trauma including traumatic brain injury and hemothorax after ATV accident
18	16-year-old with intentional drug ingestion and overdose
19	5-year-old with status asthmaticus
20	3-month-old infant with nonaccidental trauma
21	15-year-old with NMDA receptor encephalitis and altered mental status secondary to status epilepticus
22	8-year-old with viral myocarditis
23	4-year-old medically complex child with ventriculoperitoneal shunt malfunction
24	7-year-old undergoing MRI for altered mental status with hypertension and bradycardia
25	4-month-old with rhinoviral bronchiolitis and respiratory failure
26	9-year-old with mediastinal mass
27	16-year-old with spontaneous pneumothorax
28	2-month-old with RSV and impending respiratory failure
29	8-year-old with MIS-c (multisystem inflammatory syndrome related to COVID-19 infection)
30	5-year-old with status asthmaticus
31	8-year-old with supraventricular tachycardia

**Table 2.** Settings, Assets, and Adjuncts Created for the VR Simulation Laboratory

Settings:	Four-bay PICU, three-bay emergency department, MRI suite, operating room, interventional suite, single patient room
Mannequins:	Infant, child and teen with seizure, chest tube, and intubation capability
Assets created:	Sim manager with all pediatric advanced life support (PALS) waveforms and vitals active
	IV insertion capability
	Central venous line insertion capability
	Defibrillation pads available
	Intubation supplies: bag, mask, suction ETT, oral airway, laryngoscope, ventilator attachment
	Three leads on chest for monitoring (part of the scenario for learner to ask)
	Chest tube pigtail kit for scenarios with PTX
	Respiratory adjuncts: nebulizer, high flow nasal cannula, BiPAP with large and small masks, nasal and oral airways
Radiographs:	Chest x-ray (CXR) with pleural effusion, bronchiolitis, pneumothorax, pneumonia, empyema
	CXR with and without endotracheal tube in place, central venous line in place
	CXR with enlarged cardiac silhouette for dilated cardiomyopathy, pericardial tamponade
	CXR with mediastinal mass
	Abdominal x-ray (AXR) with dilated loops of bowel and obstructive pattern
	AXR with free air
Ultrasounds:	Tamponade/large pericardial effusion
	Jugular vein/carotid artery view for central venous line insertion
	Four-chamber view of poor heart function
	Large pleural effusion (both simple and complex with loculations)
	Large abdominal ascities
Computed tomography	CT head with HIE (hypoxic ischemic changes)
Magnetic resonance imaging:	Diffuse cerebral edema
	Large intraparenchymal hemorrhage
	Large posterior fossa tumor
	Large epidural hemorrhage/large subdural hemorrhage
	Obstructive hydrocephalus

For each scenario, a code leader (resident or fellow) was identified as the decision maker during the mock code. Two study members were deployed within the VR space and took turns running the simulation manager or executing code leader orders in real time. This included gathering requested assets such as the defibrillator, display of requested laboratory studies, or radiographs at key points in the scenario, and activation of 3D objects called “holocrons,” which triggered 3D recordings in VR. This created the illusion of extra resuscitation team members in VR who could perform additional tasks such as bag-mask ventilation, initiation of chest compressions, or insertion of intravenous lines.

### Logistical considerations to execute successful VR simulation

**FIG. 4.**
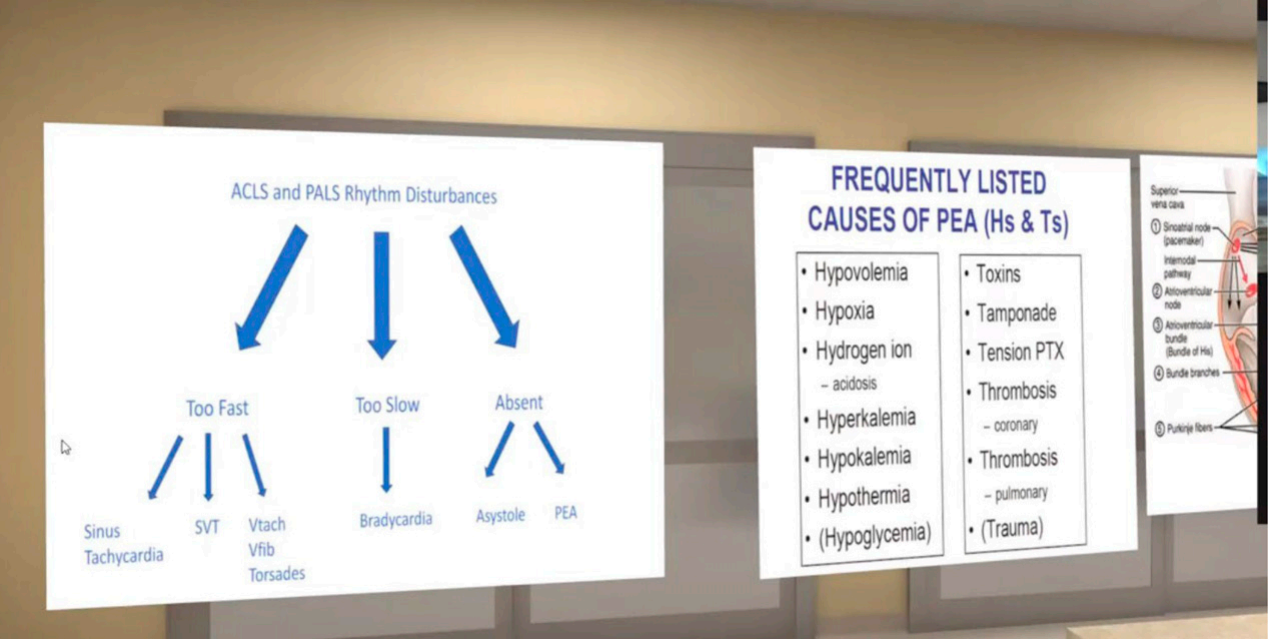
Teaching adjuncts created for the VR simulation laboratory.

The code leader and all study participants had a Zoom^®^ screen share view of the patient (Fig. 3) replicating the traditional code leader position from the foot of the bed. Participants could view the monitor, changes in vital signs, the patient, and the team members in the VR space in real time and ask in-VR study members to perform tasks such as medication administration, intubation, intravenous fluids deliver, or obtain laboratory studies. After the scenario completion, the view changed to a debriefing area where adjunctive learning aids were displayed, and the case(s) were discussed (Fig. 4).

## Results

### Participants

Thirty-two individuals participated in mock code scenarios using the VR technology between June 2021 and January 2022. The sample was 56% female and primarily PGY-2 (postgraduate year) (*n* = 28, 87.5%). Most participants were pediatric residents (*n* = 17, 53%), followed by emergency department (ED) residents (*n* = 12, 37.5%), PICU Fellows (*n* = 2, 6.25%), and combined internal medicine/pediatrics residents (*n* = 1, 3%). For analytic purposes, the PICU Fellows and the singular medicine/pediatrics resident were combined with the pediatric residents as shown in Table 3.

**Table 3.** Participant Characteristics

Participant characteristics (*n* = 32)	
Sex	
Male, *n* (%)	14 (43.7%)
Female, *n* (%)	18 (56.3%)
Training type	
ED, *n* (%)	12 (37.5%)
Pediatric	
Pediatric, *n* (%)	17 (53.1%)
PICU FELLOW, *n* (%)	2 (6.3%)
MED-PEDS, *n* (%)	1 (3.1%)
Year	
PGY-2, *n* (%)	28 (87.5%)
PGY-3, *n* (%)	2 (6.3%)
PGY-4, *n* (%)	2 (6.3%)

### Measures

A modified version of Tool for Resuscitation Assessment Using Computer Simulation (TRACS) from Brett-Fleeger et al. (2008) (Table 4) was used to evaluate the code leader for each scenario. Scoring was based on recordings of the 31 mock codes. In related studies of computer-based simulation, TRACS demonstrated good interrater reliability and showed trends toward improvement with increasing years of training, as would be expected given increased exposure to complex cases over time.^8^

**Table 4.** TRACS Edited from Brett-Fleeger 2008

Task group	Task	Y (1 pt)	N (0 pt)
	Basics		
H&P	Elicits essential information about patient and situation (attends to age, inciting event, signs/symptoms provided); solicits additional basic information such as PMH/medication		
	Performs/directs pertinent PE		
Monitors	Ensures cardiorespiratory and O2 saturation monitors placed within 1 min		
	Airway		
Assessment	Assesses breathing		
	Performs general airway maneuvers—positions patient		
	Provides any oxygen support		
Bag-valve mask ventilation	Initiates positive-pressure ventilation in timely manner		
	Correctly connects to oxygen source		
Endotracheal intubation	Initiates team efforts for endotracheal intubation in timely manner		
	Selects appropriate size endotracheal tube		
	Selects appropriate size laryngoscope blade		
	Ensures suction available		
Intubation assessment	Asks team member to auscultate patient		
	Asks for portable chest X-ray to confirm tube placement		
Airway RSI	Selects premedication(s) appropriately		
	Doses premedication(s) appropriately		
	Selects sedative/hypnotic agent(s) appropriately		
	Doses sedative/hypnotic agent(s) appropriately		
	Selects paralytic appropriately		
	Doses paralytic appropriately		
	Circulation and arrhythmias		
Basics	Assesses blood pressure (asks/looks for reading)		
CPR	Initiates appropriately		
Management	Initiates IV fluids appropriately		
Arrhythmias	Recognizes presence of abnormal (nonsinus) rhythm on initial presentation		
	Identifies first abnormal rhythm correctly		
	Doses energy correctly (may be PALS- or ACLS-based dose)		
	Time to deliver defibrillation less than 4 min from onset of ventricular fibrillation		
	Recognizes indication for first line medication (epinephrine)		
	Uses correct dose of epinephrine (0.1 cc/kg or 1 mg)		
	Repeats pulse check after CPR/resuscitation medications		
	Behavior		
Professionalism	Has professional attitude towards team members		
Leadership	Uses personnel effectively		
	Used closed loop communication effectively with team		
Management	Performs tasks in appropriate sequence/prioritizes well		
	Intermittently summarizes/ maintains global view		
	Specific learning points unique to this sim		
	Total score	35	

Code leaders scored one point for yes and zero points for no in four key categories of basics, airway, circulation, and behaviors and within subcategories, including taking a basic history, recognizing the need for positive pressure ventilation, timely CPR, recognizing arrhythmias, exhibiting a professional attitude, demonstrating leadership, and a composite for overall management. Scoring was based on careful review of the recorded Zoom^®^ session. Satisfaction with the VR training modality was assessed via an investigator-derived survey, completed online via SurveyMonkey^®^ (San Mateo, California).

### Data analysis

Independent sample *t*-tests were used to compare percentage of correct scores on the TRACS by training level (PGY-2 versus PGY-3 or above) and training type (pediatrics versus ED). Differences were not statistically significant for either comparison (*p* > 0.05) and are shown in Table 5.

**Table 5.** TRACS Results

TRACS score	Mean	SD	t	p value	Cohen’s *d*
Training type			1.082	0.288 (n.s.)	0.395
ED	0.73	0.143			
Pediatric	0.79	0.151			
Year			1.412	0.168 (n.s.)	0.755
PGY-2	0.78	0.147			
PGY-3 and 4	0.67	0.142			

Item-level satisfaction survey data were calculated to assess acceptability of this training modality. Participants rated the training modality favorably on all items. Individual comments included, “VR had a level of realism that was greater than mannequin simulation” and “VR offered more in-depth debriefing opportunities.” However, some noted that VR was not being used to its full potential because “participants could only video stream and not immerse in their own headset or perform actions themselves within the scenario.” These survey results are shown in Table 6.

**Table 6.** Survey Results

Satisfaction	Strongly disagree	Disagree	Neutral	Agree	Strongly agree	N/A
The knowledge can be applied to a real-life code situation	0.0%	0.0%	0.0%	48.7%	51.4%	
Similar learning outcomes to that of a mock code simulation using mannequins	0.0%	0.0%	10.8%	51.4%	37.8%	
VR simulation offered educational pearls not available in mock codes using mannequins	0.0%	18.9%	16.2%	32.4%	32.4%	
After VR simulation, I feel more confident about my abilities to serve as a code team leader	0.0%	2.7%	16.2%	37.8%	21.6%	21.6%
I am interested in participating in additional VR mock codes	2.7%	2.7%	5.4%	43.2%	46.0%	
I prefer VR mock code participation to traditional mock code simulation using mannequins	8.1%	13.5%	37.8%	27.0%	13.5%	

## Discussion

A commentary by Tabatabai et al. (2020) defined VR education as “instruction in a learning environment where educators and students are separated by time or space, or both, and the instructors provide course content through management applications, multimedia resources, the internet, videoconferencing, etc.”^9^ Despite some previous VR experience, application to learners in a mock code style had not yet been performed. In addition, without a VR laboratory or individual VR headsets, a combination of multimedia devices (VR plus streaming videoconferencing) needed to be used. Pandemic restrictions helped accelerate innovation and rapidly overcome administrative barriers to the use of new applications. Advances in VR training for medical students, dental students, and surgeons all followed similar patterns of innovation during this period.^10–12^

### Considerations for creation of a VR simulation center

Conventional mannequin simulation centers are the mainstay of medical team training because they allow learners to practice patient care while not putting actual patients at risk. It has been shown to reduce medical errors.^13–18^ The creation of these entities focuses on cost, space, and personnel. High-fidelity mannequins such as Laerdal’s Sim Man 3G^®^ (Stavanger, Norway), the Victoria^®^ from Gaumard (Miami, Florida), the Apollo^®^ from CAE Healthcare (Sarasota, Florida), and the Leonardo^®^ from MedVision (Arlington Heights, Illinois) range in price from $65,000 to $85,000.^19^ Mannequins can be designed to breathe, have palpable pulses, use lights to communicate patient color change associated with low oxygen levels, or allow advanced procedures such as intubation, CPR, chest tube insertion, or childbirth. The more features the mannequin possesses, the higher the price cost and needs for maintenance and repair.

Despite additional features, the level of realism remains low. A study by Sterz et al. of simulated live patients versus mannequins for emergency medicine training showed most students rated simulated patients as more realistic than mannequins. This data corroborates the results of other international studies in which interactions with simulated live patients came close to real encounters especially when noninvasive procedures or a patient interview is required.^20–23^ VR training ultimately hopes to approximate live simulated patient interaction.

Hospital systems have approached simulation centers creation from two perspectives—dedicated space versus temporary space. Dedicated simulation centers have central command stations with multiview cameras for recording, high fidelity displays, and debriefing areas. Rooms mimic different hospital settings such as operating suites or inpatient rooms with mannequins specific to the scenarios intended for that space. Dedicated space centers for simulation require significant ongoing investment on the part of a health system.

Few peer-reviewed studies detail the cost of building and maintaining a simulation program. Acero et al. reported their expense in 2012 at $3.8 million to construct the institution’s simulation center and an additional annual $1.5 million annually in operating expenses.^24^ In 2020, the University of Nebraska opened its Davis Global Center simulation training facility iEXCEL (Intraprofessional Experiential Center for Enduring Learning) program. The center houses leading-edge technology to help students understand real-life healthcare conditions and patient care techniques. Spaces include a 3D and virtual immersive reality learning studio, an electronic learning media development studio designed to deliver learning content to remote locations, a realistic simulated clinical and community healthcare space with operable systems for learning assessment, and a surgical skills simulation space. The total cost of this center was greater than $121 million.^25^

Other hospital systems choose to repurpose an existing space as a semi-permanent or temporary simulation center. Unused hospital rooms, converted wards, or communal areas are set up and then dismantled after simulation events occur. The spaces may not be patient care areas and require learners to imagine they are working on a patient in a medical setting. Room size may restrict the number of participants.

By comparison VR simulation can be surprisingly cost-effective despite concerns about high “upfront” costs.^26^ Headsets, cables, computers with advanced graphics capabilities, and partnership with a VR company are often the components that drive initial spending.^26,27^ A state-of-the-art VR headset ranges from $500 (Meta Quest 3, Menlo Park, California) to $1500 (HTC Vive Focus 3, New Taipei City, Taiwan). Headsets stand alone or link to a high-powered gaming computer such as Alienware (Dell, Miami, Florida) which ranges from $699 to $3299 and offer faster speeds, better graphics, and less image lag. VR company subscription fees allow access to existing prebuilt content as well as added content creation. For this study, given the amount of preexisting platform content and simple editing functionality, cost efficiency was maximized and centered on mannequin and waveform creation.

Owing to the sparse number of individuals who own headsets and computers, institutions may choose to build a VR laboratory where learners can borrow a headset to enter the virtual space. They may be collaborating with a person using their personal headset at home or someone in the next VR stall. At a minimum, any 6.5 by 6.5 foot area can suffice for the use of VR and owing to this limitation in space and tethering of the headset to a laptop or personal computer, movement through a large virtual environment in this study occurred by “teleportation.” Using handheld controllers, one moves from one place to another in the VR world while physically standing still. Untethered headsets can allow for greater freedom of movement through large physical environment if available.

A virtual simulation laboratory requires electricity and Wi-Fi coverage with acceptable upload and download speeds (3 megabits/second [Mbps]/10 Mbps in this project) and allows for team training and learning from multiple remote sites all while seeing and hearing each other in real time within the virtual space. In a perfect world, perhaps, each learner would have their own headset and ancillary equipment for the execution of VR simulation remotely. The creation of physical VR laboratory does add to the overall startup cost and can be thought of in the framework of traditional simulation centers. Cost considerations include procurement of a dedicated space on the hospital campus with multiple VR stalls (minimum 6.5 × 6.5 feet), laptops, and headsets, which are estimated at roughly $2500–3000 per unit, subscription to a VR platform which can range between 30 to 100,000 dollars yearly (depending on number of licenses required), fees associated with creation of new content (estimates depend on the complexity of the scenario) and fees associated with adequate staff salary and benefits. Despite these considerable fees, when compared with traditional mannequin simulation, VR startup cost and ongoing maintenance remains significantly lower.

VR simulation’s ability to stage training in multiple simulated hospital areas provides a significant advantage over traditional simulation. This study routinely held its mock code exercises in the eight-bay intensive care unit, the four-bay emergency department, the interventional radiology suite, and the postanesthesia recovery area, all of which were available for use and modification in the platform. Whitfill et al. demonstrated a substantial net decrease in costs for a digital simulation over a mannequin-based simulation for disaster triage.^27^ Although we have yet to stage a mass casualty exercise, an entire cityscape with multiple accidents and corresponding victims already exists for use.

The cost savings associated with staffing VR simulation is debatable. Software applications tout their ability to allow for standalone learning obviating the need for staff. Users can start the program and independently execute a scenario allowing them to proceed at their own pace and repeat the scenario multiple times. However, simulation staff may still be needed to troubleshoot login, password, Wi-Fi, and unexpected computer issues. An additional argument against this type of independent VR is that once a learner has experienced a scenario, it may become repetitive and of limited value.

This project chose a platform that closely replicated traditional simulation and required simulation staff. A member of the research team designed the scenario and controlled the vital signs and rhythm changes reacting to the decisions made by the residents and fellows in real time. The “simulation manager” would activate various tokens within the scenario to reveal laboratory and radiologic studies and allowed for creativity, spontaneity, and variability in each scenario. Upload and use of various learning adjuncts in a virtual debriefing area separate from the simulation itself allowed for in-depth education sessions and helped complete the exercise in a lower stress environment.

A novel facet of this project is that it addressed the lack of an established VR simulation center and paucity of learners with access to their own headsets. Within the VR product, by setting up a remote camera in the virtual space at the foot of the patient’s bed, the code leader could see the patient, the monitor, and the immediate room surroundings. This view was then screen shared via a secure Zoom link to all participants who offered suggestions to the code leader either by direct audio conversation or by typing their suggestions in the chat feature.

This novel workaround, however, created problems when scoring the mock code leader. Points were assigned to the code leader using the modified TRACS as shown in Tables 4 and 5. However, it more accurately reflected the performance of the entire team who offered insights and suggestions during the scenario. Leaders with limited mock code experience could score higher or lower depending on the members of their team. Thus, our statistical analysis does not offer any real conclusions about the success of any given individual pediatric or emergency medicine resident, in terms of mock code skill and knowledge. Future iterations could try to isolate the mock code leader but would not be reflective of real code situations where multiple practitioners in the room offer help and suggestions.

An additional limitation of this study that could be considered is the lack of standardization of the 31 cases. Each individual case was run one time and chosen by the study team to represent what we felt were “bread and butter” PICU PALS-style scenarios. Each came with variability and different potential outcomes to make the experience unique for the learning group. The study group had a concern that if standardized cases were repeated at weekly intervals, residents in the program would over time know the cases ahead of time and this would lead to bias. However, our effort to provide spontaneity and variability may have also affected the project by making some cases more difficult than others thus still affecting scoring.

The relative lack of significant haptic feedback also limits the success of VR in its current form. At this time, VR is beneficial for learning the steps involved in resuscitation and procedures associated with critically ill patients. Agasthya et al. performed a controlled trial evaluating the value of a 19-min immersive VR tutorial (interventional group) on intubating an infant mannequin. The primary endpoint (the performance accuracy measured by a checklist) did not differ between groups.^28^ However, the “feel” of caring for a patient is still evolving via development of adjuncts like gloves and suits. VR can teach the steps for procedures but cannot communicate the fine touch feedback involved with precise procedures such as laryngoscopy or central venous catheter insertion.

The participants completed a series of survey questions following their experience with VR as shown in Table 6 and rated the experience overall as favorable. We feel that the Zoom streaming version, although rated favorably, would be outperformed by the immersive headset experience and autonomous navigation of the virtual space with interactive team training. Future research should focus on this immersive experience.

There is hope that XR or “mixed reality” in which VR elements are overlayed onto a physical object (such as a mannequin) may address some of these issues. It is the hope that synergy will develop between conventional mannequin simulation and VR to take advantage of their relative strengths and offer collaborative learning experiences.

## Conclusions

Overall, despite initial fees associated with hardware and platform subscriptions when compared with traditional mannequin simulation, VR simulation can keep costs lower. VR simulation can offer a more immersive, realistic experience than traditional simulation and should be considered as an adjunctive modality to traditional simulation centers. Further research should focus on the ability of VR simulation to create measurable skill acquisition and retention of complex medical scenarios.

## Abbreviations Used

VR: Virtual reality
CPR: Cardiopulmonary resuscitation
3D: three-dimensional
PICU: pediatric intensive care unit
PALS: pediatric advanced life support
ACLS: advanced cardiac life support
BiPAP: bilevel positive airway pressure
ED: emergency department
TRACS: Tool for Resuscitation Assessment Using Computer Simulation
RSV: Respirator syncytial virus
CXR: Chest x-ray
AXR: Abdominal x-ray
ATV: all terrain vehicle
